# PLGA-based control release of Noggin blocks the premature fusion of cranial sutures caused by retinoic acid

**DOI:** 10.1007/s00253-018-9457-8

**Published:** 2018-11-03

**Authors:** Weicai Wang, Chen Zhou, Zhicai Feng, Hongyu Li, Yadong Zhang, Baicheng Bao, Bin Cai, Mu Chen, Hongzhang Huang

**Affiliations:** 1 Guanghua School of Stomatology, Hospital of Stomatology, Guangdong Provincial Key Laboratory of Stomatology, Sun Yat-sen University, 56 Lingyuanxi Road, Guangzhou, China; 2 Department of Stomatology, Shenzhen Nanshan People’s Hospital and The 6th Affiliated Hospital of Shenzhen University Health Science Center, 89 Taoyuan Road, Shenzhen, China

**Keywords:** Craniosynostosis, PLGA microsphere, BMP, Noggin

## Abstract

Craniosynostosis (CS), the premature and pathological fusion of cranial sutures, is a relatively common developmental disorder. Elucidation of the pathways involved and thus therapeutically targeting it would be promising for the prevention of CS. In the present study, we examined the role of BMP pathway in the all-trans retinoic acid (atRA)–induced CS model and tried to target the pathway in vivo via PLGA-based control release. As expected, the posterior frontal suture was found to fuse prematurely in the atRA subcutaneous injection mouse model. Further mechanism study revealed that atRA could repress the proliferation while promote the osteogenic differentiation of suture-derived mesenchymal cells (SMCs). Moreover, BMP signal pathway was found to be activated by atRA, as seen from increased expression of BMPR-2 and pSMAD1/5/9. Recombinant mouse Noggin blocked the atRA-induced enhancement of osteogenesis of SMCs in vitro. In vivo, PLGA microsphere encapsulated with Noggin significantly prevented the atRA-induced suture fusion. Collectively, these data support the hypothesis that BMP signaling is involved in retinoic acid–induced premature fusion of cranial sutures, while PLGA microsphere–based control release of Noggin emerges as a promising strategy for prevention of atRA-induced suture fusion.

## Introduction

Craniosynostosis (CS), the premature fusion of one or more of the cranial sutures, which causes secondary deformations of the cranial vault, cranial base, and brain, occurs with an estimated birth prevalence of 1 in 2000 to 2500 live births worldwide (Johnson and Wilkie 2011; Wilkie et al. 2010). Craniosynostosis can lead to dramatic clinical manifestations in terms of cosmesis and functional impairment including craniofacial deformation, mental retardation, and difficulties with vision, hearing, and breathing (Wilkie and Morriss-Kay 2001). Approximately 21% of craniosynostosis patients have pathologic genetic alterations (86% single-gene mutations and 14% chromosomal abnormalities) (Wilkie et al. 2010). Craniosynostosis has also been associated with environmental factors including fetal constraint and maternal tobacco, alcohol, and drug use (Carmichael et al. 2008; Gardner et al. 1998; Honein and Rasmussen 2000; Jentink et al. 2010; Kallen 1999; Olshan and Faustman 1989; Sanchez-Lara et al. 2010; Zeiger et al. 2002). Despite the recent progress in molecular biology and using animal models, many details of the suture biology and the pathophysiological mechanisms of craniosynostosis remain unknown.

Retinoic acid (RA), a metabolite of vitamin A, plays a key role in a variety of biological processes and is essential for normal embryonic development (Rhinn and Dolle 2012). However, retinoic acid is also a notorious teratogenic factor causing craniofacial deformations including craniosynostosis (Gardner et al. 1998). High doses of retinoic acid on prenatal *Macaca nemestrina* result in the premature formation of all coronal sutures (Yip et al. 1980). Microarray analysis showed that the level of retinol-binding protein 4 (RBP4), a transport protein for retinol in serum, was decreased 37-fold in prematurely fused/fusing sutures compared to unfused sutures (Coussens et al. 2007). RBP4 was also found to be significantly downregulated during mineralization of human cranial suture mesenchyme cells, analogous to its decrease in pathological suture fusion (Leitch et al. 2013). Moreover, human null and hypomorphic mutations in the gene encoding the RA-degrading enzyme CYP26B1 can also lead to craniosynostosis (Laue et al. 2011). Recently, RA has been shown to increase gene expression of several hedgehog and bone morphogenetic protein (BMP) ligands, which might reveal the potential mechanisms of retinoic acid-induced craniosynostosis (James et al. 2010).

BMPs play key roles in skeletal developmental patterning, including osteoblast and chondrocyte differentiation, cartilage and bone formation, and craniofacial and limb development (Rahman et al. 2015; Matsui and Klingensmith 2014; Nie et al. 2006; Norrie et al. 2014). BMPs and their inhibitors are involved in the development of normal suture patency as well as the pathological suture fusion. BMP2 was highly expressed in the cells isolated from fused sutures of sagittal and coronal synostosis patients, while BMP4 was highly expressed in those of fused metopic or lambdoid synostosis (Whitton et al. 2016). The expression of BMP4 was elevated in the craniosynostotic sutural ligament and dura of coronal craniosynostotic rabbits (Rottgers et al. 2016). Study found that enhanced BMP signaling through a constitutively active form of the BMP type 1A receptor (BMPR-1A) in cranial neural crest cells caused premature suture fusion in mice (Komatsu et al. 2013). In a genome-wide association study for nonsyndromic sagittal craniosynostosis, susceptibility loci were found near BMP2 and within BBS9 (Bardet-Biedl syndrome 9), which were genes related with skeletal development (Justice et al. 2012).

In the present study, we test the hypothesis that BMP signaling is involved in retinoic acid–induced premature fusion in vitro and in vivo to better illustrate the molecular mechanisms of retinoic acid–induced premature suture fusion. Moreover, we explored the possibility of control-released Noggin from biocompatible PLGA microspheres in prevention of all-trans retinoic acid (atRA)–induced CS.

## Materials and methods

### Specimen preparation

Newborn C57BL/6J mice were purchased from the Medical Animal Center of Sun Yat-sen University (Guangzhou, China). For normal development of the suture, the skulls of mice at postnatal day 15 (P15), P20, P25, P30, P35, P40, and P45 was harvested. For subcutaneous injection model, mice were divided into two groups: control and atRA. atRA (R2625, Sigma-Aldrich, St. Louis, USA) was diluted in ethanol at 0.01 M (3 mg/ml), and approximately 10 μl was delivered by subcutaneous injection into the area near the posterior frontal suture at P10 at a final dose of 100 mg/kg of mice weight. The skulls of the mice were harvested at P20. All skulls were fixed in 4% paraformaldehyde (AR1068, Boster, Wuhan, China) at 4 °C prior to microCT scanning.

### Micro-computed tomography scanning and reconstruction

Mouse skulls were scanned by high-resolution micro-computed tomography (microCT, Scanco Medical μCT 50, Switzerland) with scanning parameters set to 70 kV and 200 μA, a voxel size of 20 μm, and 1024 × 1024 resolution. The integration time per projection was 300 ms. During scanning, the skulls were placed vertically into a polyethylene cylinder. Reconstruction and analysis of the skulls were performed using Avizo 8.1 (FEI Visualization Sciences Group).

### Histological staining

After decalcification with 0.5 M EDTA for 1 month, the skulls were serially dehydrated with various concentrations of ethanol and xylene, embedded in paraffin, and sectioned (5 μm thick) according to standard procedures. Cross sections of the posterior frontal sutures were collected. Sections were deparaffinized and stained with hematoxylin and eosin (H&E) to examine suture development of atRA-injected and control mice.

### Cell culture

Isolation and culture of the suture-derived mesenchymal cell were performed as previously described (James et al. 2010; Xu et al. 2007). Five-day-old C57BL/6J pups (*n* = 80) were purchased from the Medical Animal Center of Sun Yat-sen University (Guangzhou, China). The pups were sacrificed by decapitation, and posterior frontal sutures with 500-μm bony margins were dissected meticulously in sterile, cold phosphate-buffered saline (PBS, pH 7.2–7.4). To minimize tissue heterogeneity, sutures were separated from the underlying dura mater and overlying pericranium. Approximately 10 sutures were placed in a 6-cm culture dish with the endocranial surface face down in 1 ml of control medium (Dulbecco’s modified Eagle’s medium with 10% fetal bovine serum, 100 U/ml penicillin, and 100 U/ml streptomycin (Gibco, USA)) at 37 °C in a 5% CO_2_ atmosphere. Medium was replenished every 2 days. First-passage suture-derived mesenchymal cells were used for all cell culture experiments. After attachment, SMCs were supplied with either control medium (CM), CM with 1 μM atRA (CR), osteogenic differentiation medium (OM, StemPro® Osteogenesis Differentiation Kit, Gibco, USA), or OM with 1 μM atRA (OR). Medium was changed every other day. RNA and protein were collected at day 14 for expression analysis.

### Cell proliferation assay

Logarithmic-phase SMCs were plated into 96-well plates at a density of 5000 cells/well in DMEM with 10% FBS, 100 U/ml penicillin, and 100 U/ml streptomycin. After 24 h of incubation, the medium was replaced with CM, CR, OM, or OR. At days 0, 1, 2, 3, 4, and 5, 10 μl of CCK-8 (Dojindo, Japan) solution was added to each well. After an additional 1 h of incubation at 37 °C, absorbance was measured at 450 nm using a microplate reader (Infinite200, Tecan).

### Alkaline phosphatase assay and staining

To determine ALP activity levels in SMCs, a colorimetric assay was performed using an ALP assay kit (Nanjingjiancheng, Jiangsu, China) according to the manufacturer’s instructions. At days 7 and 14, cells were lysed in 0.1 mol/l Triton X-100 (MP) for 1 h and centrifuged at 1000 rpm for 10 min, after which the supernatants were collected. P-Nitrophenylphosphate was used as a substrate to detect ALP activity. The optical density of the red product paranitrophenol was measured at 520 nm using a microplate reader (Infinite200, Tecan). The total protein concentration of cell lysates was measured using a BCA Protein Assay Kit (CWBio, Beijing, China). ALP activity was expressed as the release of 1 mg p-nitrophenol per 15 min per microgram of total cellular protein. On days 7 and 14, ALP staining was performed using the Alkaline Phosphatase Staining Kit (Stemgent, San Diego, USA) following the manufacturer’s guidelines. Alkaline phosphatase appeared red or purple under light microscopy.

### Alizarin red S staining

Alizarin red staining was performed to detect extracellular mineralization. Briefly, cells were fixed with 4% paraformaldehyde at room temperature for 10 min and then incubated with 1% alizarin red S (MP, USA) for 1 h, followed by extensive washing with distilled water. The staining of calcium mineral deposits of terminally differentiated cells was recorded using an inverted phase-contrast microscope (Axiovert 40, Zeiss).

### Quantitative real-time reverse transcription-polymerase chain reaction

Total RNA was extracted using Trizol reagent (Invitrogen, USA). cDNA was synthesized using a Roche RT-PCR System (Roche). Real-time reverse transcription-polymerase chain reaction (RT-PCR) was performed using two-step RT-PCR assays (Roche). Markers of osteogenic differentiation (runt-related transcription factor 2 (Runx2), alkaline phosphatase (ALP), type I collagen (Col1a1), osteopontin (OPN), and osteocalcin (OC)) and markers of BMP signaling (BMPR-1a, BMPR-1b, BMPR-2, BMP2, BMP4, Noggin, smad1, smad5, and smad9 (also known as Smad8)) were examined. The specific primers used for detecting mRNA transcripts are shown in Table 1. Transcripts were normalized according to the β-actin transcript levels and compared with the control using the 2^-ddCt^ method.

**Table 1:** Primer pairs used for RT-PCR amplifications

Gene	Primer
BMPR-1A	F: GGCCATTGCTTTGCCATTAT R: CGGTGAATCCTTGCATTGAAA
BMPR-1B	F: GGACATGCTGGACTTGGCTTC R: TTATTAGGGACTTGTGAGCCTGGAC
BMPR-2	F: GAGCCAGACGGCAAGAGCTTA R: TCGCTTCATAGTTGGAGACGAGAG
Smad1	F: GGAATGCTGTGAGTTCCCATTTG R: TGCTGAGGATTGTACTCGCTGTG
Smad5	F: AGACCATGCCCAGCATATCCA R: TTGACAACAATCCCAGGCAGAA
Smad9	F: AGGTCTGCATCAACCCATACCATTA R: ACTTCGGAACTTGGCCAGGAG
Noggin	F: GAAGTTACAGATGTGGCTGTGGTC R: AAGCAGCTGCCCACCTTCA
BMP2	F: TGACTGGATCGTGGCACCTC R: CAGAGTCTGCACTATGGCATGGTTA
BMP4	F: TTTGTTCAAGATTGGCTCCCAAG R: AAACGACCATCAGCATTCGGTTA
Runx2	F: CCAGAATGATGGTGTTGACG R: GGTTGCAAGATCATGACTAGG
ALP	F: GGGACGAATCTCAGGGTACA R: AGTAACTGGGGTCTCTCTCTTT
OPN	F: TACGACCATGAGATTGGCAGTGA R: TATAGGATCTGGGTGCAGGCTGTAA
OC	F: GGGCAATAAGGTAGTGAACAGACT R: CAAGCAGGGTTAAGCTCACACT
Col1a1	F: GACATGTTCAGCTTTGTGGACCTC R: GGGACCCTTAGGCCATTGTGTA
β-Actin	F: CATCCGTAAAGACCTCTATGCCAAC R: ATGGAGCCACCGATCCACA

### Preparation of Noggin-encapsulated microspheres with PLGA and in vivo treatment

Microspheres of poly (d-l-lactic-co-glycolic acid) (PLGA, Sigma, St. Louis, MO) of 50:50 were prepared as described before. Briefly, 250 mg PLGA was dissolved into 1 ml chloromethane, and 50 μl of recombinant Noggin (R&D Systems, Minneapolis, MN) or the control bovine serum albumin was mixed with 1 ml PLGA solution. Then, 2 ml of 1% polyvinyl alcohol (PVA) was added in PLGA solution, followed by 1-min mixing. A total of 100 ml of 2% isopropanol was added to the final emulsion and stirred for 2 h. Microspheres were stored in liquid nitrogen and lyophilized for at least 48 h before use. Control release kinetics of Noggin from the PLGA microsphere in vitro was analyzed by the ELISA kit as instructed.

### Treatment of recombinant mouse Noggin in vitro and in vivo

SMCs were cultured in OR with or without recombinant mouse Noggin (200 ng/ml, RD) for 21 days for alizarin red staining. For in vivo analysis of the efficacy of the microsphere based therapy, atRA-induced CS mice were injected with 2 mg Noggin- or BSA-encapsulated PLGA microspheres dissolved in 20 μL collagen gel 1 day post atRA injection. Mouse skulls were harvested at P20 for microCT scanning.

### Statistical analysis

All experiments were performed at least three times. All quantitative data are expressed as the mean ± standard deviation and were analyzed using Student’s *t* test or one-way ANOVA followed by a post hoc Tukey range test in GraphPad Prism, with significance set at *P* < 0.05. In figures, bars in graphs represent means, and error bars represent 1 SD.

## Results

### atRA caused premature fusion of the posterior frontal suture in a subcutaneous injection mouse model

**Fig. 1:**
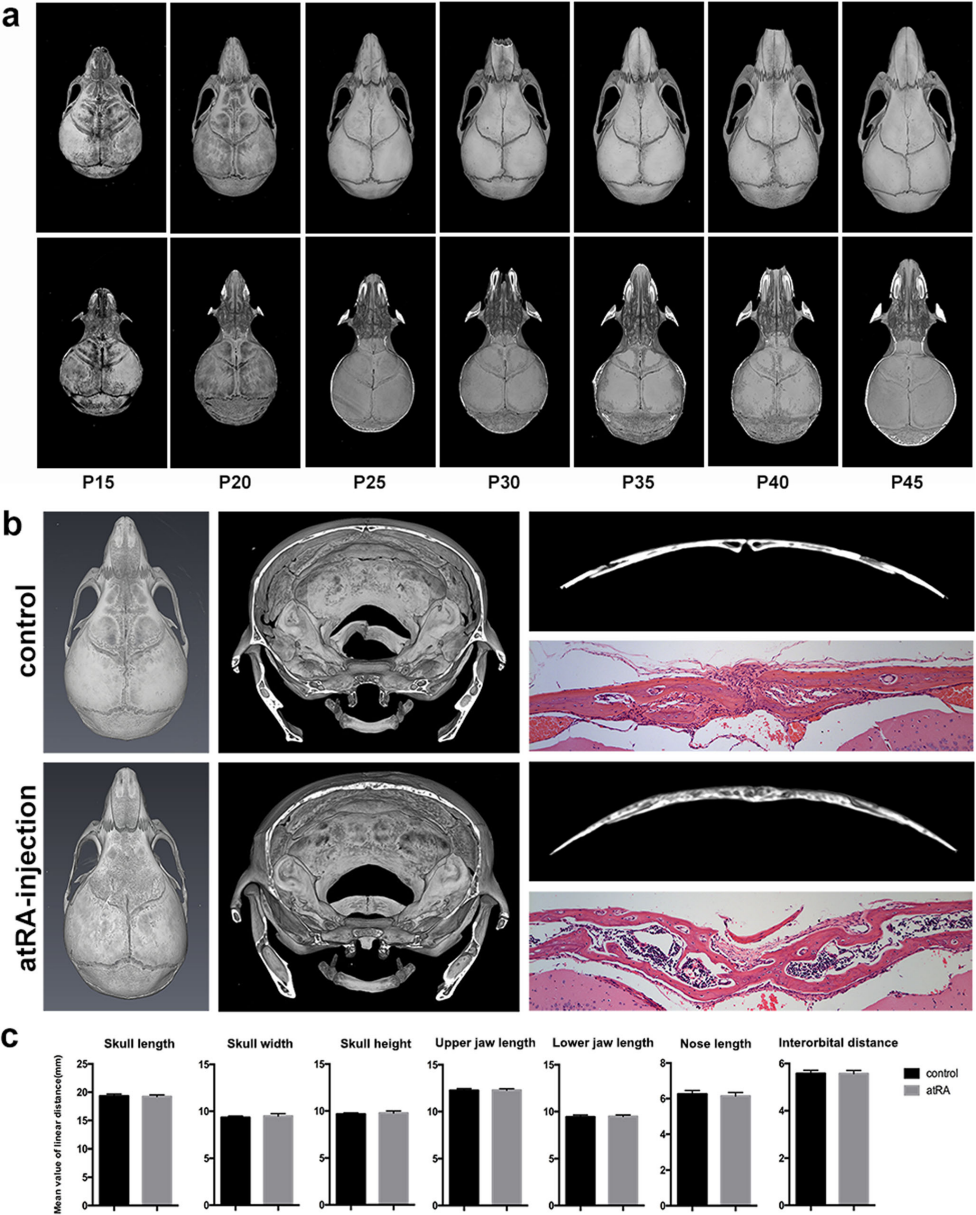
atRA caused premature fusion of posterior frontal suture in a subcutaneous injection mouse model. **a** Time course of the development of the suture in normal mice. **b** 3D reconstruction and H&E staining of the morphology of posterior frontal suture in atRA-injected and control mice. The posterior frontal sutures of P20 mice in the atRA-injected group (*n* = 6) fused prematurely, while those in control mice (*n* = 6) continued to develop normally. **c** Measurements of the skull and facial skeleton of atRA-injected and control mice. No significant differences were found in skull length, skull width, skull height, nasal length, maxilla length, mandible length, or interorbital distance between the atRA-injected mice (*n* = 6) and the control group (*n* = 6)

As the posterior frontal suture is the only calvarial suture fuses physiologically, precise course of the fusing time was analyzed from P15–P45. The posterior frontal suture fused from 20 and 45 days postnatal at the ectocranial layer from anterior to posterior, while the endocranial layer remains open till P45 (Fig. 1a). To establish a mouse model of atRA-induced craniosynostosis, atRA was injected into the area near the posterior frontal suture subcutaneously. In the subcutaneous injection assay, the posterior frontal sutures of P20 mice in the atRA-injected group (*n* = 6) fused prematurely, while those in control mice (*n* = 6) developed normally. The trabeculae in these fusion sutures were irregularly arranged in both cross sections of 3D reconstruction and H&E staining (Fig. 1b). We quantified the skull morphology of both control and atRA-injected mice using anatomical landmarks representing the entire skull and the facial skeleton. No significant differences were found in skull length, skull width, skull height, nasal length, maxilla length, mandible length, or interorbital distance between the atRA-injected mice (*n* = 6) and the control group (*n* = 6) (Fig. 1c).

### atRA repressed the proliferation of SMCs

**Fig. 2:**
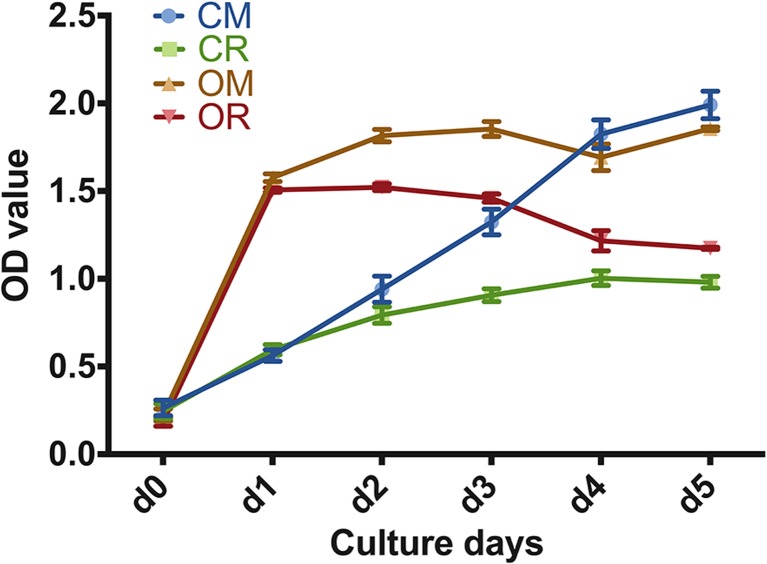
Cell proliferation of SMCs in response to different media. Osteogenic medium increased the proliferation of cells in OM and OR at day 1. atRA decreased the proliferation of cells in the CR and OR groups after day 1 when compared with the corresponding control groups (*P* < 0.05). Values represent calculated means and are normalized to culture in CM. Error bars = 1 SD; *n* = 3; *P < 0.05

To determine the effect of either atRA or osteogenic medium on SMC proliferation, cell proliferation assay was performed at days 0, 1, 2, 3, 4, and 5 (Fig. 2). The OD value increased significantly in osteogenic differentiation medium (OM and OR) compared with that in control medium (CM) at day 1, after which the OD plateaued. After atRA treatment, the OD values of cells in the CR and OR groups decreased significantly after day 1 in comparison with those in the corresponding control groups (*P* < 0.05).

### atRA enhanced in the osteogenic capacity of SMCs

**Fig. 3:**
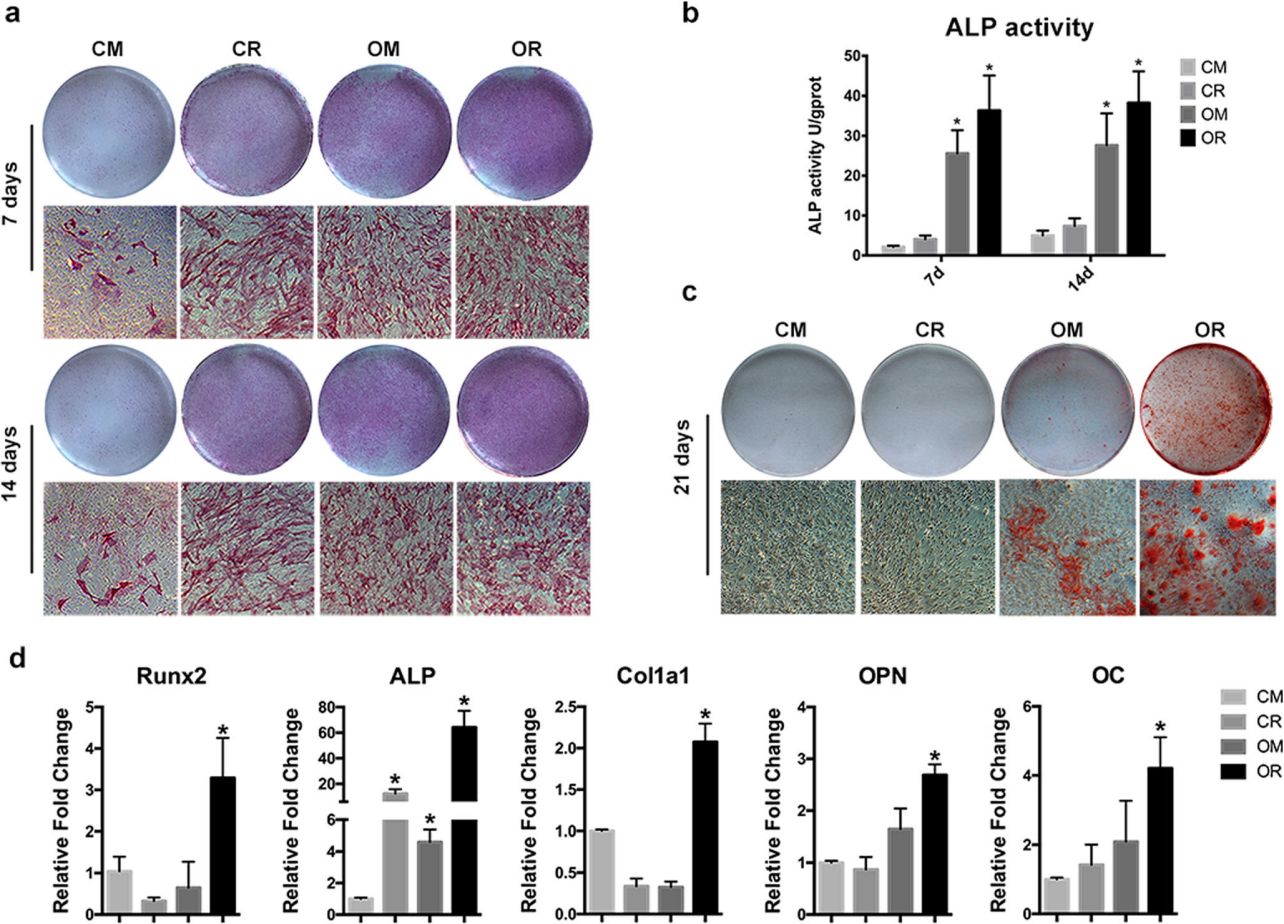
atRA increased osteogenesis in SMCs. atRA increased alkaline phosphatase staining (**a**) and alkaline phosphatase activity (**b**) at day 7 and day 14 of culture in differentiation media. **c** atRA enhanced mineralization in SMCs as measured by alizarin red S staining. **d** atRA increased the mRNA level of markers related to osteogenesis. Runx2, ALP, Col1a1, OPN, and OC were significantly upregulated at day 14 in osteogenic differentiation medium supplemented with atRA (*P* < 0.05). Values represent calculated means and are normalized to culture in CM. Error bars = 1 SD; *n* = 3; *P < 0.05

To identify the effect of either atRA or osteogenic medium on ALP activity in SMCs, ALP staining and ALP activity assay was detected at days 7 and 14. Strong ALP staining was observed in the OR groups at both day 7 and day 14, whereas the OM and CR groups demonstrated weaker alkaline phosphatase staining, and no staining was observed in the CM groups (Fig. 3a). The results of ALP activity were consistent with those of the ALP staining at day 7 and day 14. Osteogenic differentiation medium induced mesenchymal cells to exhibit increased ALP activity in OM and OR cultures at days 7 and 14 compared with the corresponding control groups in CM, whereas CR did not significantly increase ALP activity. The maximal expression level of ALP activity was observed in cells cultured in OR (*P* < 0.05), which was approximately 20- and 8-fold greater than the levels measured in the CM cultures at days 7 and 14, respectively. Comparing the ALP activity between the OM and OR groups, the OR group showed significantly higher ALP activity than the OM group at day 14 (*P* < 0.05), whereas ALP activity in OR and OM did not differ at day 7 (*P* > 0.05) (Fig. 3b). The above results suggested that osteogenic medium increased ALP activity in SMCs and that these effects were enhanced with atRA supplementation of osteogenic medium.

To detected extracellular mineralization, mineralized nodules of SMCs in the four different media were evaluated at 21 days of culture using alizarin red S staining. In OM, mineralized nodules were present at 21 days in culture, and this mineralization increased with the supplementation of atRA, whereas there were no bone nodules in CM or CR (Fig. 3c). Accordingly, several osteoblast markers, the mRNA level of Runx2, Alp, Col1a1, Opn, and Oc, were significantly upregulated at 14 days in osteogenic differentiation medium with atRA supplementation (*P* < 0.05) (Fig. 3d). In particular, Alp mRNA levels in OR were increased 60-fold compared with CM and 12-fold compared with OM.

### atRA enhanced the osteogenic capacity of SMCs via BMP-SMAD pathway

**Fig. 4:**
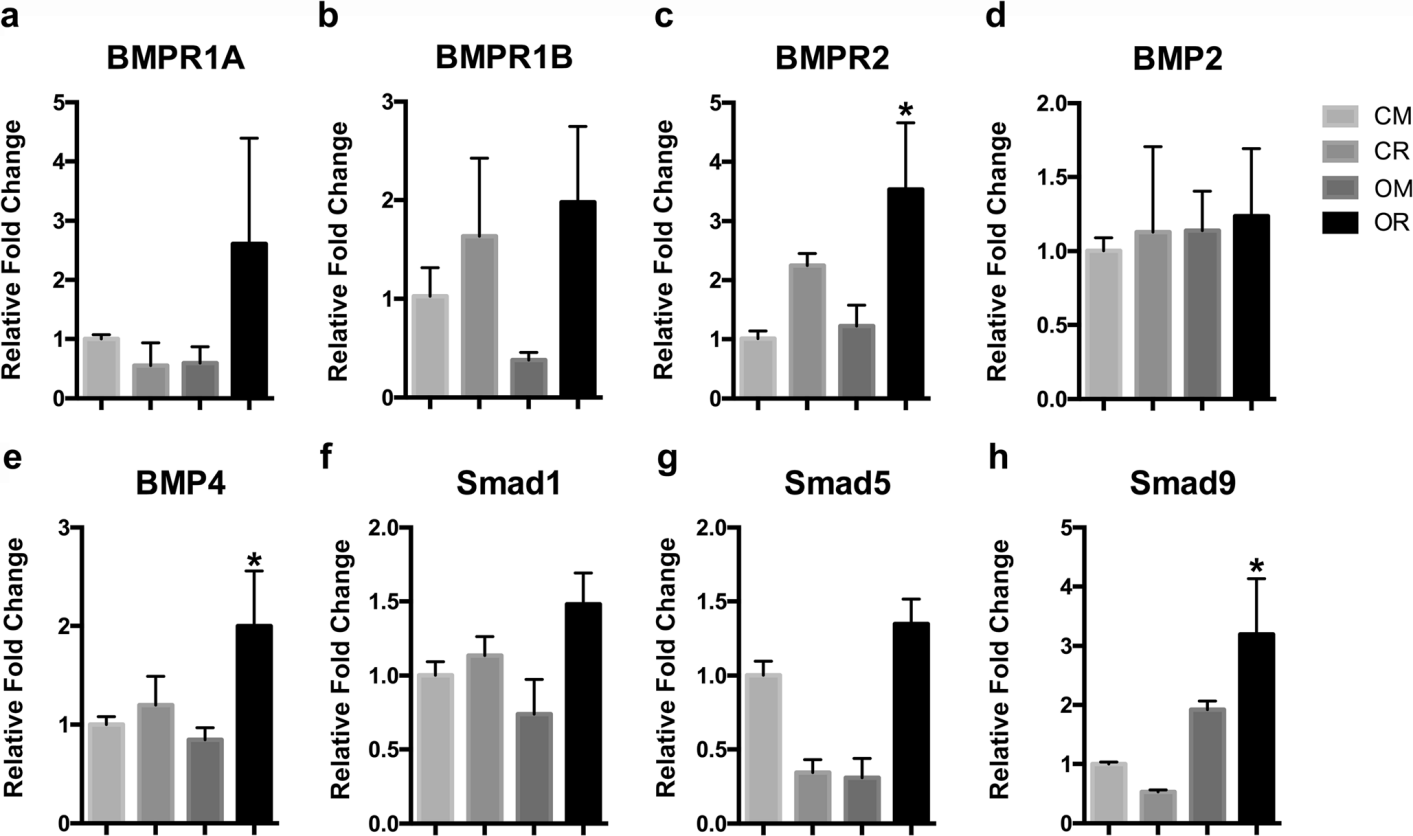
atRA increases the mRNA and protein expression of BMP signaling components in SMCs. The mRNA expression levels of Bmpr-1A (**a**), Bmpr-1B (**b**), Bmpr-2 (**c**), Bmp2 (**d**), Bmp4 (**e**), Smad1 (**f**), Smad5 (**g**), and Smad9 (**h**) in samples as indicated were analyzed by qPCR (*P* < 0.05). Error bars = 1 SD; *n* = 3; *P < 0.05

Since BMP signaling has been shown to promote osteogenic differentiation of several cell lines, we evaluated the mRNA levels of genes related to BMP signaling. The mRNA expression of Bmpr-2 was observed to be significantly (*P* < 0.05) upregulated, by approximately 3.5-fold, in cells cultured in OR compared with CM or OM at day 14, while the transcript levels of Bmpr-1a and Bmpr-1b were also increased but not significantly (*P* > 0.05) (Fig. 4a–c). The relative mRNA level of Bmp4 increased approximately 2-fold in cells cultured in OR compared with that in CM or OM, while that of Bmp2 remained unchanged (Fig. 4d, e). Smad1, Smad5, and Smad9 were measured because they are immediate downstream effectors in the BMP signaling pathway. Smad9 mRNA expression was upregulated at day 14 under OM and OR culture conditions (*P* < 0.05). However, Smad1 and Smad5 mRNA levels were only modestly upregulated in the cells cultured in OR compared to those cultured in CM (*P* > 0.05) (Fig. 4f–h). These results suggested that atRA might enhance the osteogenic differentiation of SMCs at least in part through upregulation of BMP/Smad signaling.

### PLGA microsphere–based Noggin control release blocks the atRA-induced suture fusion

**Fig. 5:**
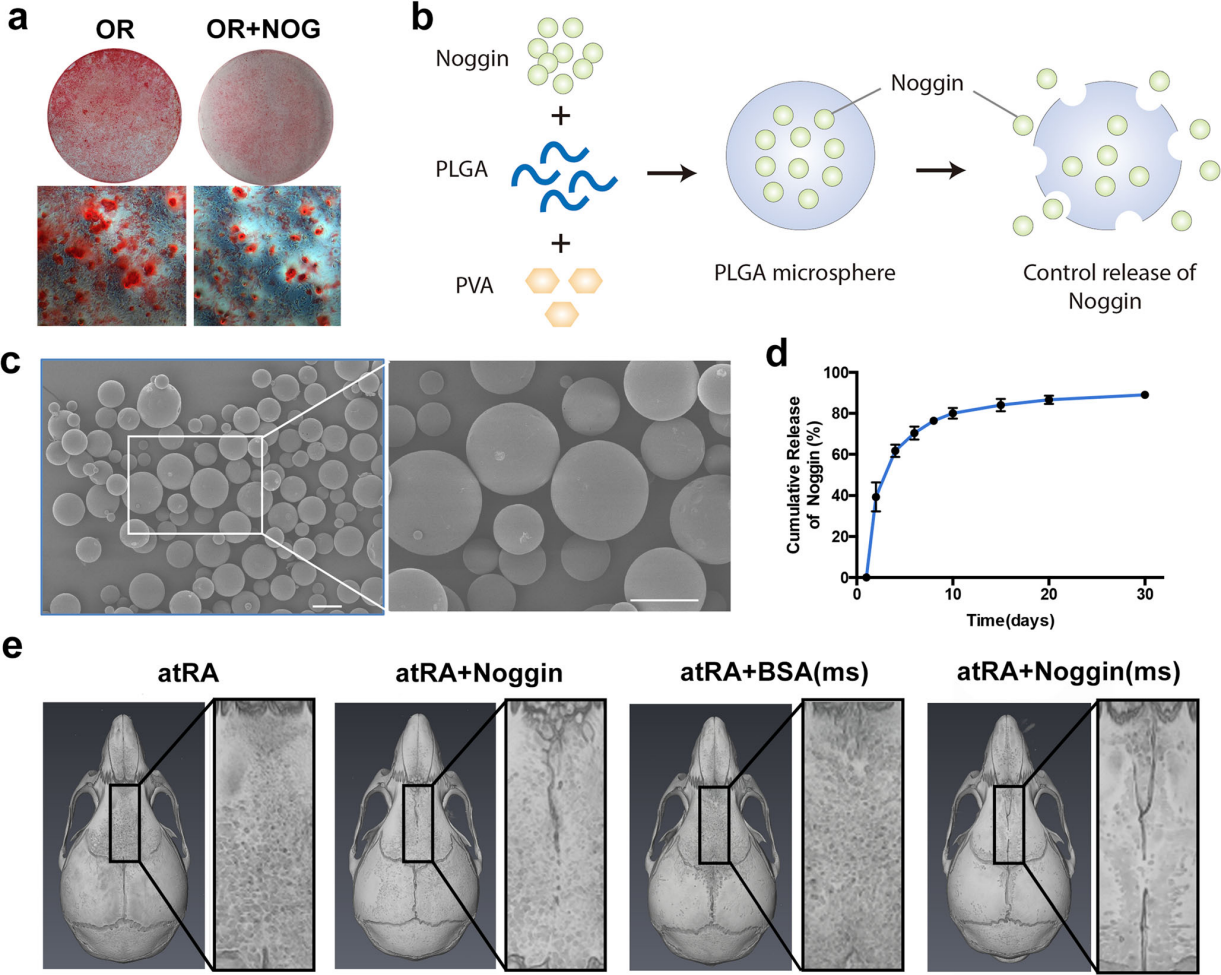
Noggin delivery blocked the atRA-induced suture fusion. **a** Recombinant mouse Noggin inhibited the atRA-induced enhancement of osteogenesis of SMCs as measured using alizarin red staining. **b** Schematic representation of Noggin encapsulation in PLGA microspheres. **c** The scanning electron microscope image of PLGA microspheres. **d** Release kinetics further confirmed the control release of Noggin from the PLGA microspheres. **e** Representative image of the fusion of the posterior frontal suture in indicated groups (*n* = 6 for each group)

Noggin inactivates the BMP signaling pathway by competing with receptors for ligand binding. To test the effects of Noggin in vitro, SMCs were cultured in OR with or without recombinant mouse Noggin. After 21 days of induction, alizarin red staining showed that recombinant mouse Noggin inhibited the atRA-induced enhancement of osteogenesis of SMCs, with many fewer mineralized nodules present in the Noggin-treated group (Fig. 5a). We then tested whether control release of Noggin would block the atRA-induced suture fusion in vivo. First, Noggin or control BSA was encapsulated into the PLGA microspheres (Fig. 5b), which was confirmed by a scanning electron microscope (Fig. 5c). Release kinetics further confirmed the control release of Noggin from the PLGA microspheres (Fig. 5d). Next, 1-day post atRA-induced CS, mice were tropically injected with 2 mg Noggin- or BSA-encapsulated microspheres dissolved in 20 μL collagen gel. As shown in Fig. 5e, the fusion of posterior frontal suture was partially blocked in Noggin-injected mice, while those injected with Noggin-encapsulated PLGA microspheres were much more obvious than those of naked Noggin.

## Discussion

Retinoic acid, a well-known teratogen, has been shown to be involved in craniosynostosis (CS). However, the mechanisms by which retinoic acid causes craniosynostosis are unclear yet. In the current study, we for the first time revealed that excessive BMP signal pathway is essential for atRA-induced CS. Moreover, we also revealed that PLGA microsphere encapsulated with Noggin, an inhibitor of BMP pathway, significantly prevented and partially restored the atRA-induced suture fusion in vivo*.*

BMPs are key growth factors in skeletal differentiation that interact with bone morphogenetic protein receptors (BMPRs) on the cell surface to phosphorylate the transcription factors Smad1/5/9, thereby allowing them to bind with Smad4; the Smad complex then activates BMP target genes (Rahman et al. 2015; Wu et al. 2016; Miyazono et al. 2010; Sieber et al. 2009). BMP signaling pathways play important roles during embryonic development and early skeletal formation, and abnormal BMP signaling can cause skeletal disorders (Bandyopadhyay et al. 2013; Li and Cao 2006; Rosen 2006; Yamamoto and Oelgeschlager 2004). Recombinant human BMP4 induced significant changes in alkaline phosphatase activity in a dose-dependent manner in cells derived from either wild-type or craniosynostotic rabbits (Cooper et al. 2010). Transgenic mice expressing a truncated dominant-negative BMPR-1B targeted to osteoblasts using the type I collagen promoter showed impairment of postnatal bone formation, and their bone mineral density, bone volume, and bone formation rate were severely reduced (Zhao et al. 2002). Mice with a conditional knockdown of BMPR-1A had wide-open anterior fontanelles and developed short faces, hypertelorism, and calvarial foramina (Saito et al. 2012). Another study showed that enhanced BMP signaling through the BMPR-1A in cranial neural crest cells caused premature suture fusion in mice (Komatsu et al. 2013). As for BMPR-2, there were controversies of the effect of BMPR-2 on the development of the skeletogenesis as well. Gamer (Gamer et al. 2011) found that loss of BMPR-2 in the early limb has no effect on limb skeletal patterning or endochondral ossification, whereas BMPR-2 mutants displayed delayed ossification in the lateral ossification centers of vertebrae and the interparietal bone (Delot et al. 2003; Yang et al. 2010). Recently, Smad9 was demonstrated to be a new type of transcriptional regulator in BMP signaling, and its expression increased when cells exposed to BMP4 (Tsukamoto et al. 2014). Our study here further revealed that the BMP-SMAD pathway is involved in atRA-induced suture fusion. Our study showed that BMP pathway genes, in particular mRNAs for BMPR-2, BMP4, and Smad9, were abundantly expressed in SMCs, which were further enhanced by atRA, suggesting that atRA might increase the osteogenic differentiation capacity of SMCs by transcriptionally regulating the BMP-SMAD pathway.

In light of the involvement of BMP-SMAD pathway, we further explored whether inhibition of BMP pathway would prevent the premature fusion. Noggin was selected based on the findings that Noggin could block BMP function by binding BMP-2, BMP-4, BMP-5, BMP-6, and BMP-7 with various degrees of affinity (Canalis et al. 2003). As expected, we revealed that control release of Noggin via PLGA microspheres significantly blocked the fusion in atRA-induced craniosynostosis in mice. The intervention experiments further concluded that atRA induces CS via BMP pathway. Moreover, the study also provided a delivery strategy and a drug candidate for CS prevention. The finding here is consistent with the previous studies on the role of Noggin. For example, Warren has found that ectopic noggin expression prevented the fusion of mouse posterior frontal sutures (Warren et al. 2003). In rabbit and rat models of coronal suture synostosis, Noggin was found to inhibit bone healing and delay resynostosis at the surgical site after suturectomy (Cooper et al. 2007; Shen et al. 2009). In a later study, Noggin was proved to be no effect on the initial suture fusion of a delayed-onset coronal suture synostosis rabbit model (Cray et al. 2011). Currently, treatments for craniosynostosis include suturectomy, minimally invasive craniectomy, and spring-mediated distraction osteogenesis (Gasparini et al. 2012; Okada and Gosain 2012); however, there are high rates of mortality and resynostosis in surgery, and distraction osteogenesis has limited effects on the cranial vault (Hermann et al. 2013). The proposed control release of Noggin via PLGA microspheres might be useful therapeutic strategy for rescuing atRA-induced craniosynostosis.

It is important to note the timing of suture fusion observed in our study was slightly different from that in previous studies. In our animal model, we found that the posterior frontal suture fused from 20 and 45 days postnatal at the ectocranial layer from anterior to posterior, while the endocranial layer remains open till P45. The difference might be explained by that the different strains of mice have variation in the timing of suture fusion. In fact, the calvarial sutures fuse in a specific time sequence. For example, in humans, the metopic suture begins to fuse after the first year, while the other sutures begin to fuse in adulthood (Rice 2008). In the mouse, the posterior frontal suture fuses at 25–45 days of age, while the other calvarial sutures remain patent through life (Bradley et al. 1996). Recent study found that the endocranial layer of PF suture fusion in CD1 wild-type mice occurs from approximately postnatal days 7 to 15, while the ectocranial layer remains patent until P25 (Sahar et al. 2005).

In summary, we here revealed that excessive BMP signal pathway is essential for atRA-induced CS, while PLGA microsphere encapsulated with Noggin significantly prevented and partially restored the atRA-induced suture fusion in vivo. These data suggest that PLGA microsphere–based control release of Noggin emerges as a promising strategy for prevention of CS related to atRA-induced suture fusion.
